# Semi-Annual Report of the House Surgeon of the “Buffalo Hospital of the Sisters of Charity”

**Published:** 1856-06

**Authors:** 


*House Surgeon and Physician's Semi-Annual Report to the Board of Physicians and Surgeons, and Staff, of the "Buffalo Hospital of the Sisters of Charity," from Oct.* 1, 1855, *to April* 1, 1856.

ATTENDING

| *Physicians.* | | *Surgeons.* |
|---|---|---|
| Dr. Rochester, | | Dr. Hamilton, |
| " Hunt. | | " Boardman. |
| | *Marine Ward.* | |
| Dr. Wilcox, | | Dr. Samo. |

BENJ. H. LEMON,
House Surgeon.

| | |
|---|---|
| Whole number treated, was | 487 |
| There were males, | 374 |
| Females, | 113 |
| Adults, | 389 |
| Under twenty-one years of age, | 98 |
| Discharged cured, | 268 |
| " improved, | 105 |
| *Died*—Males, | 16 |
| Females, | 6 |
| Remaining, | 92 |

*Ages.*—5 years, 4; 5 to 10, 14; 10 to 15, 17; 15 to 20, 56; 20 to 25, 129; 25 to 30, 114; 30 to 40, 95; 40 to 50, 31; 50 to 60, 15; 60 to 70, 7; 70 to 80, 5.

*Nativities.*—Canada, 7; England, 24; France, 13; Germany, 144; Ireland, 216; Italy, 2; Prussia, 5; Switzerland, 5; Scotland, 8; U. States, 63.

*Diseases of those that Died.*—Ascites, 1; Delirium Tremens, 1; Enteritis, 2; Typhoid Fever, 4; Typhus Fever, 1; Gangrena-Pulmonum, 1; Morbus Cordis, 1; Hæmatemesis, 1; Meningitis, 1; Paralysis, 3; Phthisis, 3; Pneumonitis, Double, 1; Tabes Mesenterica, 1; Morbus Brightii, 1. Mortality, per centage, about five.

Of the diseases treated, it will, perhaps, suffice, if a brief list of those of the more grave character is appended, as follows:

Ascites,

Abscess, Pelvic and others,

Burns, by melted iron and camphene,

Colica Pictonum,

Cirrhosis of Liver,

Cystitis,

Coxalgia,

Delirium Tremens,

Dislocation,

Enteritis,

Empyema,

Epilepsia,

Erysipelas,

Fever, Intermittent,

" " Pernic,

" Typhus,

" Typhoid,

Fractures, Compound,

" Comp. Commin,

" Comp. Com. Comp.,

Fistulæ,

Frost-bite,

Gangrena Pulmon.,

Hæmatemesis,

Hernia,

Hæmoptysis,

Labium Leporum,

Lithotomy,

Meningitis,

Morbus Cordis,

" Uteri,

Necrosis,

Opthalmia,

Paralysis,

Phthisis,

Phlebitis,

Pneumonia,

Purpura,

Schirrus,

Scalds,

Scrophula,

Syphilis,

Ulcers,

Wounds, Gun shot, and others,

&c., &c., &c.

Of Fevers there were 140 cases, including all types; five died, one with typhus and four with typhoid.

Chirrhosis of Liver, one case, recovered.

In twenty cases of Fracture, there were several in each class, four were of the cervix femoris in aged women.

One of Gangrene of the Lungs, fatal.

Several of Heart Disease, one fatal, in which the heart weighed 46 oz.

Meningitis, one case, fatal.

Paralysis, four cases, three fatal.

Phthisis, nineteen cases, three fatal.

Phlebitis, three cases, recovered, one a female.

Pneumonia, eleven cases, one fatal, (double) in a female, after typhoid fever.

Purpura, one case, recovered.

Schirrous, four cases — pharynx, mammæ, axillary glands and pylorus.

A number of cases of Syphilis, secondary and tertiary.

